# Characteristics of Porous Starch from Lotus Seeds Using Dextranase: Protection and Sustained Release of Proanthocyanidins

**DOI:** 10.3390/foods14061050

**Published:** 2025-03-19

**Authors:** Yuying Wang, Ming’ao Wang, Weihong Jiang, Siying Li, Siyu Liu, Mingwang Liu, Mingsheng Lyu, Shujun Wang

**Affiliations:** 1 Jiangsu Key Laboratory of Marine Bioresources and Environment/Jiangsu Key Laboratory of Marine Biotechnology, Jiangsu Ocean University, Lianyungang 222005, China; yuyingw@jou.edu.cn (Y.W.); mingaowang@126.com (M.W.); weihongjiang@jou.edu.cn (W.J.); syingli@jou.edu.cn (S.L.); syliu2025@163.com (S.L.); mwliu@jou.edu.cn (M.L.); mslyu@jou.edu.cn (M.L.); 2 Co-Innovation Center of Jiangsu Marine Bio-Industry Technology, Jiangsu Ocean University, Lianyungang 222005, China

**Keywords:** Dextranase, porous starch, proanthocyanidins, sustained release

## Abstract

Porous starch, known for its large specific surface area due to internal pores, exhibits excellent adsorption capabilities. In this study, we successfully produced porous starch from lotus seeds using dextranase and conducted a comprehensive analysis of its surface morphology, crystalline structure, pasting behavior, and adsorption characteristics. The enzymatic treatment resulted in the development of a pore structure on the lotus seed starch (LS) surface without altering its crystalline structure, as confirmed by Fourier transform infrared spectroscopy and X-ray diffraction. The oil and water absorption capacities of the porous starch increased by 14% and 27%, respectively. Differential scanning calorimetry indicated a higher pasting temperature for the porous starch. This starch exhibited remarkable drug-carrying capabilities, absorbing up to 18.23 mg/g of proanthocyanidins and significantly shielding them from UV damage. In vitro release tests in simulated intestinal fluid revealed that the encapsulated proanthocyanidins (PC) achieved nearly complete release. These results underscore the potential of LS as a drug carrier and provide valuable insights for developing innovative intestinal drug delivery systems.

## 1. Introduction

Lotus seeds are nutrient-rich, providing a significant source of proteins and carbohydrates, which can be beneficial for human health [1]. Studies have shown that the high concentration of linear amylose in lotus seed starch (LS) imparts resistance to digestion, making it a suitable material for encapsulating active substances or probiotics. Despite its potential, the application of natural starch as an adsorbent is restricted due to its low specific surface area and pore volume [2]. Consequently, porous starch (PS) has garnered interest for its remarkable adsorption properties. Its unique porous structure lends it multiple functionalities and a broad spectrum of applications [3]. As a natural polymer material with a porous configuration, PS serves as both an adsorption medium and a carrier in the food and pharmaceutical industries, capable of effectively adsorbing and encapsulating bioactive components [4]. For instance, Cao et al. [5] developed a porous structure in glutinous corn starch using a combined approach of α-amylase and glucoamylase, enhancing its efficiency as a naringenin carrier. Similarly, Li et al. [6] prepared PS from loquat kernels via ultrasound-assisted acid/enzymatic hydrolysis to load metal palladium, exploring the differences in palladium loading capacity and thermal stability between conventional starch and PS. In summary, PS has a wide range of potential applications in drug delivery systems [7]. Its distinctive structural features and functional benefits allow it to act as a carrier for the slow release of drugs, potentially increasing drug effectiveness and safety [8]. In addition, the protective effect of PS can shield drugs from negative external environmental factors like light and oxidation, thereby improving drug stability [9].

Proanthocyanidins (PC), a group of polyphenolic compounds, have been widely researched for their numerous health benefits and biological properties. These compounds are particularly known for their exceptional antioxidant abilities [10], which effectively eliminate superoxide anion radicals and hydroxyl radicals [11] and protect against damage induced by oxidative stress [12]. As natural antioxidants, PCs also exhibit immunosuppressive and anti-allergic effects, which may be beneficial in preventing allergic reactions [13]. Furthermore, PCs have been shown to enhance memory, decelerate aging, and decrease the risk of stroke [14]. Their anti-inflammatory qualities also position PCs as potential inhibitors of inflammatory responses, which could help in reducing the incidence of chronic diseases [15]. However, PCs are highly susceptible to oxidation when exposed to air during processing or storage, which can reduce their stability. Therefore, it is crucial to employ special adsorption media to preserve the integrity of PCs [16].

In this study, we challenged the conventional approach of using multi-enzyme synergism (e.g., α-amylase and saccharase) or acid-enzyme co-processing by pioneering a dextranase-specific modification technique for the preparation of lotus seed porous starch (LPS). Compared to traditional methods, this innovative strategy selectively hydrolyzes α-1,6 glycosidic bonds within starch granules, optimizing the pore structure while preserving the high linearity of lotus starch. This approach not only effectively addresses the limitations of natural starch, such as small specific surface area and insufficient adsorption sites, but also significantly reduces energy consumption and process complexity by employing glucoamylase alone, aligning with the principles of green biomanufacturing. The resulting LPS was successfully utilized for the loading and protection of proanthocyanidins, with comprehensive characterization studies conducted on the formed complexes. From both the perspective of enzymatic mechanism innovation and carrier function validation, this study confirmed the potential of porous starch-polyphenol complexes for targeted intestinal drug delivery, offering a valuable reference for the development of novel intestinal drug delivery systems.

## 2. Materials and Methods

### 2.1. Materials

Dextranase was produced from *Bacillus* sp. QN6 after fermentation and concentration (enzyme activity 1.8 U/mL). LS was purchased from Yiyang Dafu Forest Foods Bio-technology Co., Ltd., located in Yiyang, China. Proanthocyanidins, amyloglucosidase (from *Aspergillus niger*, 100,000 U/mL), pancreatin (130,000 U/g), and vanillin were obtained from Shanghai McLean Biochemical Technology Co., in Shanghai, China. Reagents such as 3,5-dinitrosalicylic acid, sodium chloride, sodium hydroxide, sodium bicarbonate, and methanol were purchased from Shanghai Sinopharm Reagent Co., also in Shanghai, China. Pepsin (from swine stomach, 30,000 U/g) was procured from Dulai Biotechnology Co. in Nanjing, China, and α-amylase (5000 U/g) was supplied by Merck Life Sciences Ltd. in Nantong, China. All other chemicals used were of analytical grade.

### 2.2. Methods

#### 2.2.1. Preparation and Characterizations of LPS

Preparation of LPS

Two grams of LS were accurately weighed and mixed with 10.0 mL of Tris-HCl (pH 7.5, 0.1 mol/L) buffer. After vortex shaking and sonication (120 W) for 5 min, 50.0 mL of dextranase (enzyme activity 1.8 U/mL, optimal activity condition: 45 °C, pH 7.5) was added. The mixture was then placed on a shaking bed at 35 °C with a speed of 180 rpm for 24 h for enzyme digestion. To terminate the reaction, 5 mL of 95% ethanol was added, followed by centrifugation at 4000× *g* for 5 min. After the reaction was stopped by the addition of ethanol, the mixture was centrifuged again at 6000× *g* for 5 min, washed with distilled water 2–3 times, and centrifuged to remove the supernatant. The precipitate was then dried in a vacuum oven for 12 h, ground, and stored at 4 °C for further observation and analysis.

Determination of dextranase activity

The enzyme activity was determined by the 3,5-dinitrosalicylic acid (DNS) method. The substrate (75 μL of Tris-HCl buffer with pH 8 + 75 μL of 3% dextran T20) and 50 μL of enzyme were mixed in a tube and placed in a water bath at 45 °C for 20 min. Then, 200 µL of DNS was added and set in boiling water for 5 min. Three milliliters of distilled water was added, and the solution was detected at OD_540 nm_. In the control group, DNS was mixed with the substrate, and then the enzyme solution was added. The amount of enzyme required to release 1 μmol of glucose per minute was regarded as one enzyme activity unit (U).

Observation of morphological characteristics of LPS

Lotus starch treated with dextranase for various durations was evenly spread onto the surface of conductive adhesive, coated with gold, and then examined using a scanning electron microscope (SEM, Regulus 8230, Hitachi, Tokyo, Japan) at a magnification of 5000 times.

Particle size distribution

The particle size of LPS was measured by a nanometer particle size potentiometer (Zetasizer Nano ZS90, Malvern, Shanghai, China). The cuvette used was a disposable sizing cuvette, and the samples were suspended in ultrapure water at a concentration of 0.01% (*m*/*v*). The refractive indices used were 1.33 for the dispersant and 1.53 for the sample. Each sample underwent three scans at a temperature of 25 °C.

Determination of oil/water absorption rate

The oil and water absorption rates were measured with slight modifications to the method described by Purwitasari [17]. Two hundred milligrams each of LS and LPS were weighed into separate centrifuge tubes of known weight. Two milliliters of either rapeseed oil or distilled water were added to the respective samples and vortexed for 10 min. The mixtures were then allowed to stand at room temperature for 30 min before being centrifuged at 4000× *g* for 10 min. After centrifugation, the upper layer of liquid was decanted, and the tubes were inverted until no more oil or water dripped out. The oil and water absorption rates, expressed as percentages (O for oil and W for water), were calculated according to Equation (1).

(1) $$O/W\;(\%)=\frac{W_{2}-W_{1}-W_{0}}{W_{1}}\times 100$$

where W_0_ is the mass/g of the centrifuge tube; W_1_ is the dry mass/g of LPS; W_2_ is the mass/g of starch in the centrifuge tube after centrifugation for oil/water absorption.

Differential scanning calorimetry (DSC)

According to the method of Luo [18] with slight modification, 3 mg of each LS and LPS sample was combined with 9 μL of distilled water in a ratio of 1:3 within an aluminum crucible. The crucible was sealed and stored at 4 °C for 12 h to allow moisture equilibration. The samples were then heated from 25 °C to 120 °C at a rate of 10 °C/min, using an empty crucible as the reference. Analysis was conducted to determine the start temperature (*T_o_*), peak temperature (*T_p_*), and end temperature (*T_c_*) using the software associated with the instrument. The heat flow curve was further integrated over the range of phase change temperatures. After integrating to obtain the area, it is calculated using the following equation:

(2) $$\Delta H(J/g)=\frac{Q}{n}$$

where ΔH is the enthalpy thermal change. Q is the area under the heat flow curve obtained by integration (J), and n is the mass of the sample (g).

#### 2.2.2. Adsorption of PC by LPS

Plotting of the PC standard curve

The content of PC was determined by adapting the vanillin-hydrochloric acid method originally used by Nakamura et al. [19]. PC was used as a standard, and 240 mg of PC was accurately weighed. This amount was then dissolved in ultrapure water to prepare a standard solution with a concentration of 4.8 mg/g of PC, which was subsequently diluted stepwise to achieve a series of concentrations of 0.4, 0.8, 1.2, 1.6, and 2 mg/mL, respectively. Then, 1 mL from each concentration was transferred into a 10 mL centrifuge tube. To each tube, 3 mL of 1% vanillin-methanol solution and 1 mL of concentrated hydrochloric acid were added, thoroughly mixed, and then the mixture was allowed to react for 30 min at 30 °C in an incubator, shielded from light. The absorbance was measured at 500 nm using ultrapure water as a blank control. A standard curve was constructed by plotting PC concentration on the horizontal axis and absorbance on the vertical axis.

Determination of loading capacity

Ten milligrams of PC were weighed and dissolved in 10 mL of ultrapure water. LPS was then gradually added to the PC solution, followed by vortex mixing and sonication for 15 min. The mixture was placed in a constant-temperature incubator at 37 °C for 30 min to allow for sufficient adsorption. Afterward, it was centrifuged for 10 min at 4000× *g*, the supernatant was discarded, and the precipitate was dried and set aside. 1 mL of the PC solution, both before and after adsorption, was pipetted into a 10 mL centrifuge tube. The absorbance value at 500 nm was measured using the hydrochloric acid-vanillin method. The concentration of PC in the solution was calculated based on the previously plotted standard curve, and the PC loading of PC-LPS was calculated according to Equation (3).

(3) $$\mathrm{PC}\;\mathrm{loading}(m/g)=\frac{C_{0}-C_{1}}{M}\times V$$

In Equation (3), C_0_ is the initial concentration of the PC solution (mg/mL), C_1_ is the concentration of PC after adsorption (mg/mL), V is the volume of supernatant (mL), and M is the mass of LPS (g).

#### 2.2.3. PC Adsorption Conditions

Adsorption affected by PC concentration

The enzymatic LPS-24 h was accurately weighed, and 5 mL of standard PC solution at different concentrations was added to achieve a ratio of LPS to PC of 50:1 (*w*/*w*). Each mixture was subjected to vortex mixing and shaking, followed by ultrasonication for 15 min. Afterward, each mixture was centrifuged for 10 min. The content of PC in the initial solution and the supernatant after adsorption was determined, and the amount of PC adsorbed was calculated using Equation (3). The precipitate was then placed in an oven set at 40 °C for drying.

Adsorption affected by temperature

A PC solution with a concentration of 1.6 mg/mL was prepared, and a specified amount of LPS was accurately weighed and ultrasonicated for 15 min to form a homogeneous suspension. The adsorption experiments were conducted at various temperatures to explore how the adsorption of PC by LPS varied with temperature changes.

#### 2.2.4. Fourier Transform Infrared Spectroscopy (FTIR) of Samples

The dried LPS, PC-LPS, and PC were mixed with potassium bromide in a ratio of 1:200 and milled into a powder. The mixture was then pressed at a pressure of 10 Pa to form a sheet approximately 4 mm thick to ensure successful transmission of the light source. The wave number range was set between 4000–400 cm^−1^ with a resolution of 1 cm^−1^, and a total of 32 scans were conducted.

#### 2.2.5. X-Ray Diffraction (XRD) of Samples

An appropriate amount of dried LPS, PC-LPS, and PC was placed into the center groove of a slide and pressed down using a flat, smooth glass plate to ensure a flat surface by scraping away any excess powder. The prepared samples were then positioned on the sample stage of an X-ray diffractometer (X’Pert3 Powder, Malvern Panalytical, Almelo, The Netherlands). The door of the instrument was closed, and the samples were scanned at a rate of 5° per minute over a range of 5 to 35° (2θ) [20]. The crystallinity of LS before and after enzymatic digestion was calculated using Equation (4).

(4) $$\mathrm{Crystallinity}\;(\%)=\frac{I_{g}-I_{n}}{I_{g}}\times 100$$

In Equation (4), I_g_ denotes the total diffraction intensity and I_n_ denotes the amorphous phase diffraction intensity.

#### 2.2.6. UV Radiation Stability of PC-LPS

The stability of PC under long-term UV irradiation was assessed as it tended to degrade and sometimes completely degrade. The method of Hao [21] was used to determine the UV radiation stability of PC-LPS. An exact amount of the PC-LPS was taken to ensure that its PC loading was equal to that of PC. Both PC-LPS and PC were exposed to UV light (wavelength 253.7 nm) on an ultra-clean bench for varying durations, with samples collected at 0, 120, 240, and 360 min. These samples were dissolved in ultrapure water and reacted with DPPH (1,1-diphenyl-2-picrylhydrazil) in the dark for 30 min. The absorbance changes were recorded at 517 nm, and the free radical scavenging rate was calculated using Equation (5).

(5) $$\mathrm{DPPH}\;\mathrm{scavenging}\;\mathrm{rate}\;(\%)=(1-\frac{A_{1}-A_{2}}{A_{0}})\times 100$$

In Equation (5), A_0_ is the absorbance value after mixing of ultrapure water + DPPH; A_1_ is the absorbance value after PC-LPS/PC + DPPH reaction; A_2_ is the absorbance value after mixing of ultrapure water + PC-LPS/PC.

#### 2.2.7. Adsorption Kinetics of PC

The primary goal of the kinetic studies on PC adsorption by LPS was to assess the adsorption efficiency and kinetic characteristics of LPS when used as an adsorbent for PC [22]. In the experiments, 200 mg of LPS was mixed with a PC solution at a concentration of 1.5 mg/g under controlled conditions of 35 °C and 180 rpm. Samples were collected periodically, and the PC release was measured following centrifugation. After each measurement, the sampled solution was returned to the system to maintain its integrity. Adsorption kinetic curves were then plotted, and the data were analyzed using adsorption kinetic models, such as the pseudo-primary or pseudo-secondary kinetic models. The pseudo-primary dynamics can be expressed as Equation (6).

(6) $$Q_{t}=Q_{e}(1-e^{-k_{1}t})$$

where Q_t_ denotes the amount adsorbed at time t (mg/g); Q_e_ is the amount adsorbed when the adsorption process reaches equilibrium (mg/g), k_1_ is the pseudo-primary adsorption rate constant, and t is the time (min).

A common form of pseudo-secondary kinetic modelling is:

(7) $$\frac{t}{Q_{t}}=\frac{1}{k_{2}Q_{e}^{2}}+\frac{t}{Q_{e}}$$

where Q_t_ is the amount adsorbed at time t (mg/g); Q_e_ is the amount adsorbed when the adsorption process reaches equilibrium (mg/g); k_2_ is the pseudo-secondary adsorption rate constant; and *t* is the time (min).

#### 2.2.8. In Vitro Slow Release of PCs

Preparation of artificial gastric and intestinal fluids

Artificial gastric and intestinal fluids were prepared with minor modifications to the method described by Jiang [23].

Artificial gastric fluid was concocted by dissolving NaCl (438.75 mg), NaHCO_3_ (15.75 mg), and pepsin (13.5 mg) in 50 mL of distilled water. The pH was then adjusted to 1.20 ± 0.01 using HCl. Artificial intestinal fluid was created by dissolving pancreatin (281.0 mg), α-amylase (200 mg), and amyloglucosidase (500 μL) in 50 mL of distilled water. The pH was adjusted to 6.80 ± 0.01 using 1 M NaOH.

Simulated gastric phase digestion

150 mg of PC-LPS (loaded with 17 mg/g) was weighed and added to 10 mL of artificial gastric fluid. The pH of the mixture was measured and adjusted to 1.2 ± 0.01 using HCl solution. This mixture was then placed in a constant-temperature shaker set at 37 °C and 120 rpm for incubation. At various intervals (0, 30, 60, 90, 120, 180, 240, 300, 360, 420 min), a 0.5 mL sample was collected from the mixture. To each sample, 0.5 mL of anhydrous ethanol was added, and an equivalent amount of artificial gastric fluid was replenished to maintain the constant volume of the gastric phase system. The content of PC in the supernatant was then determined using the vanillin-hydrochloric acid method.

Simulated enteric phase digestion

200 mg of PC-LPS was weighed and added to 15 mL of artificial gastric fluid. The pH of the mixture was measured and adjusted to 1.20 ± 0.01 using HCl solution. The sample was then incubated at 37 °C and 120 rpm in a constant-temperature shaker for 2 h before being centrifuged to isolate the precipitate. The precipitate was subsequently introduced into 10 mL of artificial intestinal fluid. The pH was measured and adjusted to 6.80 ± 0.01 using NaOH solution, and the sample was incubated at 37 °C and 120 rpm in a constant-temperature shaker. At various intervals, 0.5 mL of the mixture was collected, and 0.5 mL of anhydrous ethanol was added. An equal volume of artificial intestinal fluid was replenished to maintain the total volume of the intestinal phase solution. The PC content in the supernatant was determined using the vanillin-hydrochloric acid method.

Calculation of cumulative drug release rate

The release rate of the complex in the gastric and intestinal phases was calculated using Equation (8).

(8) $$\mathrm{Cumulative}\;\mathrm{release}\;\mathrm{rate}\;(\%)=\frac{{V_{0}C}_{n}+V_{1}\sum C_{n-1}}{D\times M}$$

In Equation (8), V_0_ simulates the initial volume of the release solution (mL); n is the number of displacements; V_1_ is the volume of the displacement solution taken up (mL); M is the mass of PC-LPS (g); and D is the PC loading (mg/g).

#### 2.2.9. Data Analysis

In each group, three replicate measurements were performed, and the experimental data were analyzed for significant differences (*p* < 0.05) using SPSS.26 software. The results were then graphed using Origin software (2018, 64-bit version).

## 3. Results and Discussion

### 3.1. Characterization of (LPS)

#### 3.1.1. Scanning Electron Microscopy (SEM) of LPS

**Figure 1:**
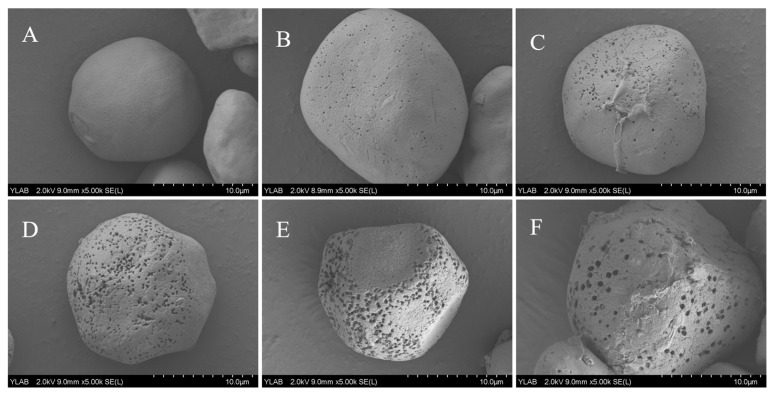
SEM images of LPS at different stages of enzymatic digestion: (**A**) LS (untreated); (**B**) LPS after 6 h digestion; (**C**) LPS after 12 h digestion; (**D**) LPS after 18 h digestion; (**E**,**F**) LPS after 24 h digestion (all images at 5000× magnification).

SEM is widely used to observe and analyze the morphology, structure, and composition of sample microregions, offering advantages such as high resolution, good depth of field, and ease of operation [24]. As shown in Figure 1A, the surface of LS appeared smooth and pore-free. In contrast, Figure 1B illustrates that after 6 h of enzymatic digestion, fine pores began to form on the surface of LPS. Figure 1C,D show that with prolonged enzymatic digestion, the pores became denser and extended into the interior of the particles, leading to a rougher surface. SEM images of LPS-24 h, shown in Figure 1E,F, reveal partial collapse of the granule surface. This phenomenon may be attributed to the hydrolysis of branched starch in the amorphous, non-crystalline region, which is relatively loose and thus preferentially degraded by the enzyme [25]. This gradual enzymatic process ultimately led to the formation of a porous structure within the starch granules, significantly enhancing the adsorption capacity of LS. Furthermore, enzymatic treatment increased the content of straight-chain starch, promoting the development of a denser crystalline structure [26]. This enhanced crystallinity improved resistance to enzymatic degradation, thereby increasing the resistant starch content. As a result, enzymatic modification not only improves the adsorption properties of LS but also enhances its potential as a resistant starch, making it highly valuable for applications in fields such as food and medicine.

#### 3.1.2. Particle Size Distribution

**Figure 2:**
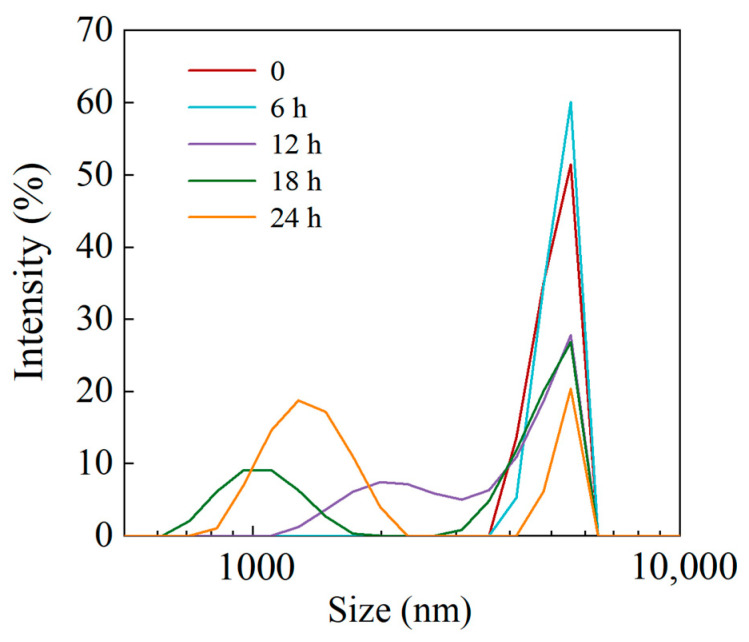
Particle size distribution of LS and LPS.

Figure 2 illustrates the particle size distribution of LS and LPS. It can be observed that after 6 h of enzymatic digestion, the starch particle size distribution remained largely unchanged compared to the original sample. However, as the enzymatic digestion time increased, a peak began to emerge below 2000 nm in the LPS particle size distribution, indicating partial hydrolysis of the starch by the enzyme. Despite this, some LS particles remained unhydrolyzed, as evidenced by a persistent peak at the same position as the original LS sample.

#### 3.1.3. Oil/Water Absorption Rate of LPS

The oil and water absorption rates of LPS are presented in Table 1. After 24 h of enzymatic hydrolysis, the oil absorption rate of LS increased significantly by 14%, while the water absorption rate rose by 27%. These findings align with those reported by Jiang et al. [27]. This improvement was likely due to enzymatic digestion altering the microstructure of LS, creating more pores. This structural modification enhanced the contact area of LPS with oil and water, thereby increasing its absorption capacity [28].

#### 3.1.4. Thermal Properties of Samples

**Figure 3:**
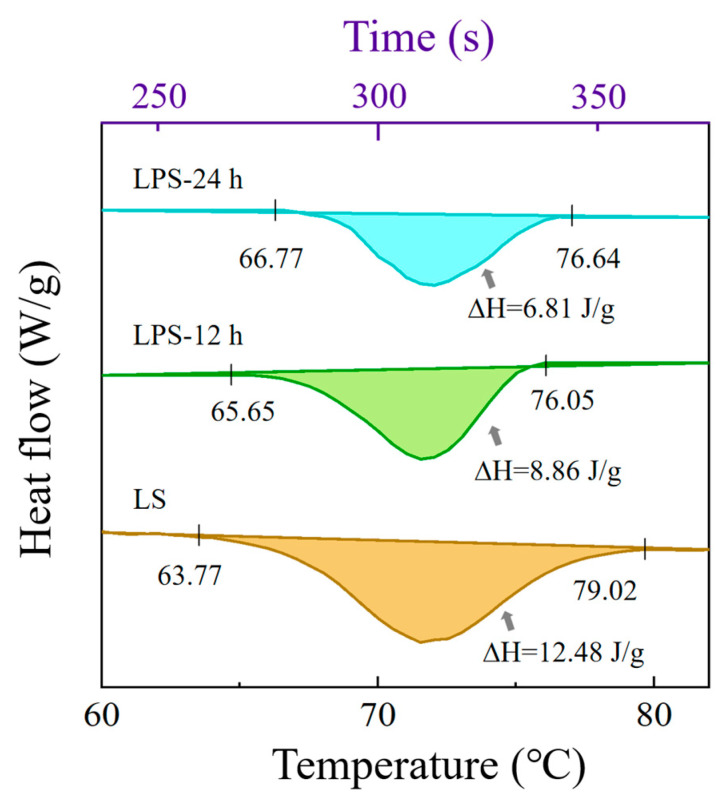
DSC of LS and LPS.

As shown in Figure 3, the pasting temperature of LPS was delayed, and the pasting temperature was higher compared to LS. This indicates that enzymatic treatment altered the thermal properties of the starch, delaying the pasting process. It is speculated that this effect resulted from a reduction in the amorphous region and an increase in the proportion of the crystalline region in LS due to enzymatic degradation. These changes likely enhanced the structural stability and resistance to pasting of the starch.

As shown in Table 2, the peak temperature (*T_p_*) of both LS and LPS exceeds 70 °C. Additionally, results from subsequent experiments indicate that this type of starch belongs to the A-type crystalline structure, which is part of the monoclinic crystalline system. In this structure, the short-branched chains of amylopectin form a dense double-helix arrangement, stabilized by a strong hydrogen bonding network [29]. This compact crystalline structure requires overcoming stronger intermolecular forces during pasting, which typically results in higher Tp values. Moreover, LPS exhibits a higher onset pasting temperature (*T_o_*) and a lower enthalpy change (∆*H*), suggesting modifications in its thermal and gelatinization properties, similar to the experimental results of Xie [30] et al. The ∆*H* represents the amount of heat absorbed or released during a chemical reaction or physical transformation [31]. The significantly lower ∆*H* of LPS compared to LS may be attributed to a reduced proportion of the double-helix structure in the branched starch molecules of LPS, which requires less energy during the pasting process [32]. This result further supports the hypothesis that enzymatic digestion increases the proportion of crystalline regions in starch granules. In general, a higher pasting temperature typically correlates with a greater degree of crystallinity [33]. The higher pasting temperature and lower enthalpy of LPS suggest better heat resistance for various applications and reduced energy consumption during the pasting process [34].

### 3.2. Adsorption of PC by LPS

#### 3.2.1. Effect of PC Concentration on Adsorption Amount

The results in Table 3 indicate that as the mass concentration of PC increased, the frequency of molecular collisions between PC and LPS also increased. This allowed more PC molecules to penetrate the pores of LPS, enhancing the interaction and thereby improving the adsorption capacity of LPS. The drug loading of LPS increased correspondingly, reaching its maximum at a PC concentration of 1.6 mg/mL, which was near the adsorption saturation point of LPS. Beyond this concentration, no significant increase in adsorption was observed.

#### 3.2.2. Effect of Different Temperatures on Adsorption Capacity

As depicted in Table 4, the PC loading increased significantly as the temperature rose within the range of 30–40 °C. This indicates that the increase in temperature enhances the mobility of the molecules and improves the adsorption efficiency of PC, thus increasing the drug loading. However, when the temperature exceeded 40 °C, PC loading began to decline. The decrease was likely due to several factors. First, excessive heat may cause the kinetic energy of molecules to surpass the binding energy threshold between the carrier and the active ingredient, leading to desorption. Second, high temperatures could potentially disrupt the porous network structure of LPS, reducing its effective specific surface area. Additionally, the thermal stability of PC may be compromised at elevated temperatures, weakening its specific binding ability to the carrier and ultimately lowering the loading capacity.

### 3.3. Characteristics of Samples

**Figure 4:**
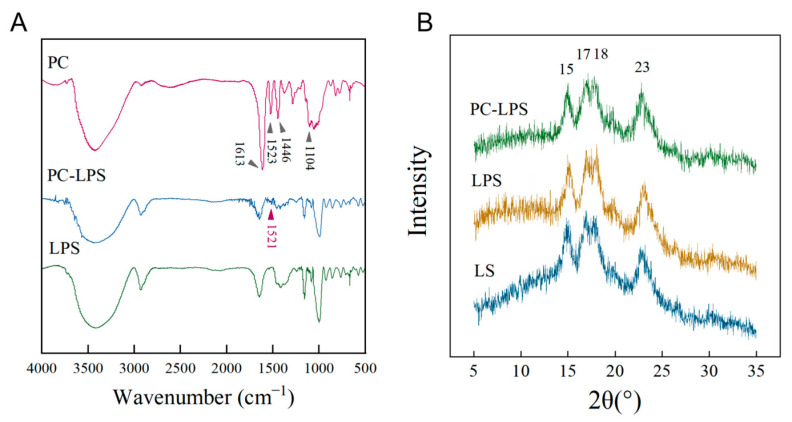
(**A**) FTIR of LPS, PC-LPS, and PC; (**B**) XRD of LS, LPS, and PC-LPS.

The results of Fourier Transform Infrared Spectroscopy (FTIR), illustrated in Figure 4A, revealed that PC exhibited notable absorption peaks at 1613, 1523, 1446, and 1104 cm^−1^, corresponding to the vibrational peaks of the aromatic ring and confirming the presence of the benzene ring structure [35]. A prominent absorption peak at 3418 cm^−1^ was primarily due to the telescopic vibration of the hydroxyl group (-OH) in the phenolic compounds of PC [36]. The absorption peak at 1613 cm^−1^ could be attributed to the characteristic functional groups of polyphenolic compounds, while the 1104 cm^−1^ peak may be linked to the out-of-plane bending vibration of the aromatic ring’s C-H, a distinctive feature of aromatic compounds [37]. Additionally, peaks at 1523 and 1446 cm^−1^ could be associated with the N-H stretching of amine and amide II and the C-N stretching vibration of amide III, respectively [38]. In comparison with LPS, the PC-LPS complex retained the original LPS characteristic peaks and introduced a new peak at 1521 cm^−1^, indicating successful PC loading. This addition not only confirms the integration of PC but may also influence the mechanical strength or chemical properties of PC-LPS.

XRD results displayed in Figure 4B shown that LS, LPS, and PC-LPS show distinct characteristic peaks at diffraction angles of 15, 17, 18, and 23°. These peaks confirm that LS possesses an A-type crystal structure [39]. Additionally, the positions of the peaks for LPS and PC-LPS complexes align with those of natural starch, indicating that the enzymatic processing and PC adsorption have not altered the crystalline structure of the starch. Additionally, the positions of the peaks for LPS and PC-LPS complexes align with those of natural starch, indicating that the enzymatic processing and PC adsorption have not altered the crystalline structure of the starch.

Further analysis of the XRD patterns was performed to determine the area of the non-crystalline regions, with the findings presented in Table 5. This analysis revealed a 13% increase in the crystallinity of the porous LS following enzymatic treatment, suggesting that enzymatic activity predominantly occurs in the amorphous regions of the starch, while its impact on the crystalline regions is minimal. This results in enhanced crystallinity of the starch post-enzymatic dissolution. Additionally, Table 5 indicates a reduction in the total diffraction intensity of LPS, likely due to a decrease in starch particle size from enzymatic hydrolysis. This reduction in size prevents the starch granules from being considered ideal crystals with infinite polycrystalline surfaces, thereby intensifying the X-ray dispersion and diminishing the intensities of the diffraction peaks [40]. The observed decrease in crystallinity of the complexes compared to LPS is attributed to PC being loaded into the amorphous regions of starch, thus affecting the overall crystallinity of the complexes [41]. These insights are crucial for understanding the interaction mechanisms between PC and LPS.

### 3.4. Adsorption Kinetics of PC

**Figure 5:**
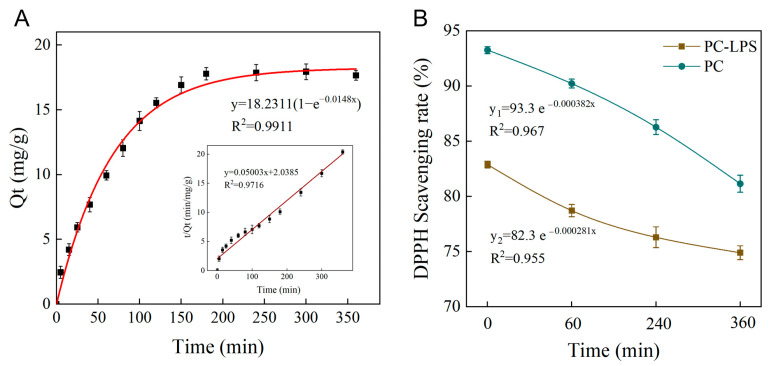
(**A**) Adsorption kinetic model for PC adsorption by LPS; (**B**) DPPH radical scavenging rates of PC and PC-LPS.

The main plot depicts the pseudo-primary kinetic model, while the inset illustrates the pseudo-secondary kinetic model (Figure 5A). The correlation coefficients for the two models were 0.9911 (pseudo-primary) and 0.9716 (pseudo-secondary), indicating that both effectively describe the adsorption behavior. However, the equilibrium adsorption value predicted by the pseudo-primary model (*Q_e_*_1_ = 18.23 mg/g) was closer to the actual experimental value (17.65 mg/g), whereas the pseudo-secondary model predicted a slightly higher *Q_e_*_2_ = 20.05 mg/g. Therefore, the pseudo-first-order kinetic model provides a more accurate representation of the adsorption behavior of PC on LPS.

Given the excellent fit of the pseudo-primary kinetic model to the adsorption behavior, it can be inferred that the main driving force of the adsorption process originates from weak intermolecular interactions. Specifically, these weak interactions—such as hydrophobic interactions, van der Waals forces, and dynamic hydrogen bonding—are characterized by a fast response and moderate reversibility, aligning well with the pseudo-primary model, which describes rapid initial adsorption followed by a gradual approach to equilibrium [42]. Further analysis, considering the saturation effect of adsorption sites, suggests that the adsorption process occurs in two distinct stages: Initial rapid adsorption: High-affinity sites on the LPS surface—such as functional groups at pore edges—are preferentially occupied by PC molecules, resulting in a steep rise in the kinetic curve. Then, the slowed adsorption phase: As surface adsorption sites become saturated, the adsorption rate is increasingly limited by molecular migration ability. At this stage, PC molecules must either penetrate the macroporous structure of LPS for bulk diffusion or overcome spatial site-blocking effects caused by previously adsorbed molecules. This constraint leads to a marked decrease in the adsorption rate, eventually reaching equilibrium. The process is consistent with the exponential decay law of the pseudo-primary kinetic model. In a related study by Lv et al. [43], a porous pasted corn starch was developed, and its ability to adsorb grapeseed porous starch (GSP) was evaluated. The results indicated that the quasi-primary kinetic model precisely described the adsorption process of porous pasted starch on GSP, with an R² value greater than 0.995.

### 3.5. UV Radiation Stability of PC-LPS

The porous structure of the starch provides a compartment for the encapsulation of proanthocyanidins, offering not only storage but also protection from environmental damage. As shown in Figure 5B, from the exponential decay equations, it is evident that the R² values for both LPS-PC and PC are close to 1, indicating a strong fit. However, the decay constants differ, with *k*_1_ = 0.000382 for PC and *k*_2_ = 0.000281 for LPS-PC. Since a larger decay constant (*k*) corresponds to a faster degradation rate, PC undergoes more rapid clearance compared to LPS-PC. This significant difference in stability highlights the protective advantage of LPS as a carrier, attributed to the spatial site resistance effect. The dense structure of the composite carrier effectively reduces the likelihood of PC molecules coming into direct contact with the external environment, thereby inhibiting photo-oxidative decomposition and thermal degradation of the active ingredients [44].

### 3.6. Slow Release of PC In Vitro

**Figure 6:**
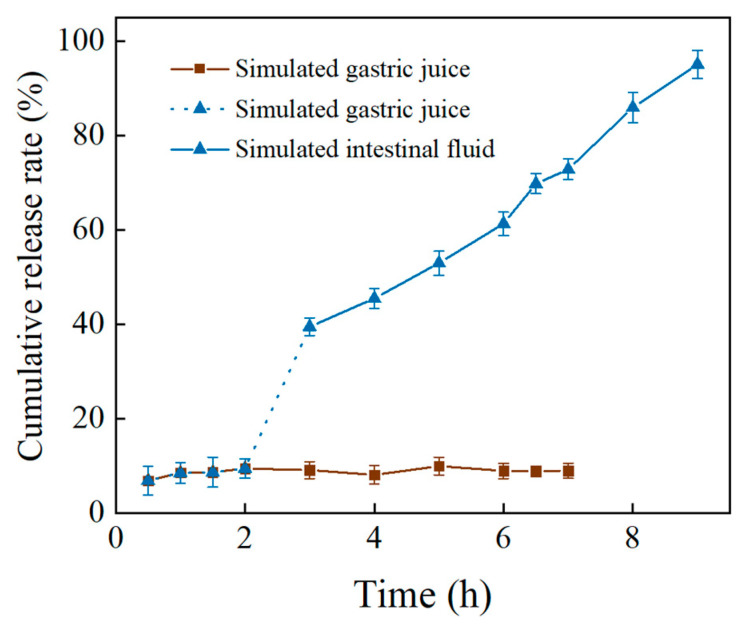
In vitro slow release of PC-LPS.

As illustrated in Figure 6, the slow release rate of PC in the stomach could be due to the resistance of LPS to the acidic environment and the digestive enzymes there, making PC-LPS more stable in acidic gastric conditions, or indicating that the gastric fluid plays a limited role in promoting the release of PC. The cumulative release of the drug in the intestinal fluid increased significantly over time, from approximately 40% at 1 h to near complete release at 7 h.

This suggests that PC can be effectively released in the intestinal fluid, likely due to the alkaline environment of the intestine, which may weaken the physical interactions (e.g., van der Waals forces and hydrophobic interactions) between LPS and PC, thereby accelerating the desorption process. Additionally, intestinal fluid contains α-amylase and amyloglucosidase, which can hydrolyze LPS, leading to the swelling of its porous structure or pore opening, thereby facilitating the diffusion and release of PC. Furthermore, the combined effects of increased pH and enzymatic hydrolysis may contribute to the structural swelling or decomposition of LPS, further enhancing the release of proanthocyanidins [45]. Chen et al. [46] conducted a study using a dynamic in vitro human gastrointestinal digestive system to examine the digestive properties of lotus seed starch-epigallocatechin gallate (EGCG) complexes (LS-EGCG) produced by various processing techniques. They found that untreated physical mixtures released almost no EGCG during gastric digestion but showed increased release during the early stages of intestinal digestion, indicating that specific processing conditions enhance the binding of LS to EGCG.

The release behavior of PC in intestinal fluid proved to be significantly superior to that in gastric fluid, potentially due to its pH sensitivity [47]. This characteristic is crucial for pharmaceutical design, especially since PC has been shown to possess anti-inflammatory properties, making it highly beneficial for creating controlled-release or intestinal-targeted drug formulations aimed at maximizing PC’s effectiveness in the intestinal tract. Consequently, innovative drug delivery systems incorporating the encapsulation of PS could be developed to fully exploit PC’s therapeutic potential in treating inflammatory bowel diseases [48].

## 4. Conclusions

LPS was prepared using dextranase. SEM analysis revealed that enzymatic treatment significantly altered the microstructure of lotus seed powder, creating a porous morphology, while the crystal structure of the starch remained largely unaffected, which makes LPS an ideal and safe drug carrier. The maximum adsorption capacity of PC by LPS reached 18.23 mg/g, and structural stability after loading was confirmed through FTIR/XRD analysis. Moreover, LPS significantly enhanced the UV stability of PC, making it a protective carrier for photosensitive drugs, effectively extending the shelf life of formulations. In vitro release experiments demonstrated a controlled PC release rate of less than 10% in simulated gastric fluid, while the cumulative release rate in simulated intestinal fluid reached 90%. This “gastric protection–intestinal release” property presents a novel strategy for developing oral intestinal-targeted drug delivery systems. Looking ahead, optimizing the particle size and pore structure of LPS carriers could enable the precise design of drug release rates, laying a technical foundation for personalized drug delivery.

This study not only establishes a scientific foundation for porous starch in drug and food delivery systems but also provides an experimental basis for understanding its interaction mechanisms with various bioactive compounds. Future research should focus on exploring in vivo efficacy, including pharmacokinetics and targeting efficiency, while evaluating release behavior in dynamic gastrointestinal environments. Further optimization of carrier formulations (e.g., polymer/liposome combinations) could lead to the development of multifunctional delivery systems. Additionally, in-depth studies on biodegradability, long-term safety, and processing stability will accelerate commercialization in pharmaceutical excipients and functional food additives, offering interdisciplinary support for precision medicine and smart nutrition.

## Figures and Tables

**Table 1:** Oil/water absorption rate of LPS.

Enzyme Digestion Time (h)	Oil Absorption (%)	Water Absorption (%)
0	78.44 ± 1.34 ^b^	81.92 ± 1.56 ^b^
24	92.68 ± 1.09 ^a^	108.49 ± 1.06 ^a^

Note: All values are means ± standard deviation; ^a,b^ within the same group indicate significant differences (*p* < 0.05).

**Table 2:** Thermodynamic properties of LS and LPS.

Samples	T_o_ (°C)	T_p_ (°C)	T_c_ (°C)	∆H (J/g)
LS	63.77 ± 0.28 ^c^	71.59 ± 0.38 ^a^	79.02 ± 0.40 ^a^	12.48 ± 0.47 ^a^
LPS-20 h	65.65 ± 0.25 ^b^	71.84 ± 0.21 ^a^	76.05 ± 0.33 ^b^	8.86 ± 0.35 ^b^
LPS-24 h	66.77 ± 0.24 ^a^	71.98 ± 0.57 ^a^	76.64 ± 0.38 ^ab^	6.81 ± 0.50 ^c^

Note: All values are means ± standard deviation; ^a–c^ within the same group indicate significant differences (*p* < 0.05).

**Table 3:** Impact of varying concentrations on the adsorption capacity of PC.

PC Concentration (mg/mL)	PC Loading (mg/g)
0.4	5.28 ± 2.31 ^d^
0.8	7.17 ± 1.18 ^c^
1.2	8.62 ± 1.36 ^b^
1.6	10.24 ± 5.27 ^a^
2.0	10.02 ± 3.15 ^a^

Note: All values are means ± standard deviation; ^a–d^ within the same group indicate significant differences (*p* < 0.05).

**Table 4:** Effect of temperature on the adsorption of PCs.

Temperature (°C)	PC Loading (mg/g)
30	9.11 ± 2.45 ^d^
40	15.94 ± 1.57 ^a^
50	12.82 ± 3.12 ^b^
55	10.37 ± 1.29 ^c^

Note: All values are means ± standard deviation; ^a–d^ within the same group indicate significant differences (*p* < 0.05).

**Table 5:** Calculation of crystallinity.

Starch Type	Crystallinity (%)
LS	36.30
LPS	49.32
PC-LPS	43.26

## Data Availability

The original contributions presented in this study are included in the article. Further inquiries can be directed to the corresponding author.
